# A Novel Probe-to-Probe Method for Measuring Thermal Conductivity of Individual Electrospun Nanofibers

**DOI:** 10.3390/ma13225220

**Published:** 2020-11-19

**Authors:** Nicholas Bonatt, John Carlin, Fangqi Chen, Yanpei Tian, Yi Zheng

**Affiliations:** 1 United States Naval Undersea Warfare Center, Newport, RI 02841, USA; nicholasbonatt@gmail.com; 2 Greystone, North Providence, RI 02911, USA; jpcarlin@greyst.com; 3 Department of Mechanical and Industrial Engineering, Northeastern University, Boston, MA 02115, USA; chen.fangq@northeastern.edu (F.C.); tian.yan@northeastern.edu (Y.T.); 4 Department of Electrical and Computer Engineering, Northeastern University, Boston, MA 02115, USA

**Keywords:** thermal conductivity, nanofibers, Probe-to-Probe method

## Abstract

Polymer nanofibers have the ability to replace expensive materials, such as metals, ceramics and composites, in specific areas, such as heat exchangers, energy storage and biomedical applications. These properties have caused polymer nanofibers to be explored as solutions to a growing list of thermal management problems, driving an even greater need to better measure and understand the thermal properties of these nanofibers. This study intends to further the understanding of the thermal properties of polymer nanofibers through the use of a novel Probe-to-Probe measurement method. Polycaprolactone nanofibers fabricated using the electrospinning method can be easily collected and loaded into a traditional atomic force microscope through a mechanical design for thermal measurement. This Probe-to-Probe method demonstrates the ability to accurately measure the thermal boundary conditions about a polymer nanofiber with a heating prong temperature up to 400 ${}^{\circ }$C and assists in characterizing its thermal properties.

## 1. Introduction

Nowadays, polymeric materials have many desirable applications in industry and everyday life. Compared with metals and ceramics, polymers are low cost and easy to fabricate. One important issue with polymeric materials is the low thermal conductivity, which limits their use for wide engineering applications. Bulk polymeric materials are usually regarded as thermal insulators with low thermal conductivity on the order of 0.1 W/(m·K) [1]. The reason for this low thermal conductivity is that there exist many defects, such as polymer chain entanglement, voids, impurities, in the bulk material. The typical method for improving the thermal conductivity is using composite materials. It has been shown that the additives, such as carbon nanotubes or metallic nanoparticles, embedded in polymer matrices [2,3], can enhance the thermal transport. However, the conductivity improvement for polymer carbon nanotube composites is restricted to one order of magnitude because of the high thermal interface resistance between the additives and the polymer matrices [4].

As the demand for small technological devices drives component sizes down to the nanometer scale, a greater understanding of thermal transport of nanoscale devices and individual nanostructures arise [5,6]. Novel thermal properties arise in low-dimensional nanostructures versus in bulk materials. Properties such as abnormal heat conduction, size dependence of thermal conductivity, phonon boundary internal and edge scatterings effect these one-dimensional structures [7,8]. Nonmetallic systems, such as polymers, transport heat via phonons [9]. While these phonons span a broad range of frequencies, those with mean-free paths <100 nm at room temperature are typically the main contributors to thermal conduction [5]. However, structures within this scale no longer adequately follow the thermal transport for bulk models [10,11]. It has been found through many studies that polymer nanofibers with highly aligned polymer chains can have significantly higher thermal conductivity and Young’s modulus than their typical bulk values [5,12,13,14,15,16]. Wang et al. used the time-domain thermoreflectance to measure the axial thermal conductivity of a single polymer fiber, and liquid crystalline PBO fibers have the highest room temperature thermal conductivity around 20 W/(m·K) as well as a Young’s modulus much higher than typical bulk values [16]. Theoretical study predicts a thermal conductivity as high as 350 W/(m·K) for a simple polymer chain of polyethylene (PE) by molecular dynamics simulations [17]. Shen et al. conducted an experiment to measure an ultrahigh thermal conductivity of PE nanofibers up to 104 W/(m·K), which is greater than the conductivities of about half of the pure metals [12]. With their thermal conductivity directly proportional to the degree of crystallinity [18,19,20], nanofiber fabrication techniques such as draw and electro-spinning are used to enhance polymer chain alignment and thus thermal conductivity [17,21,22,23,24,25].

**Figure 1:**
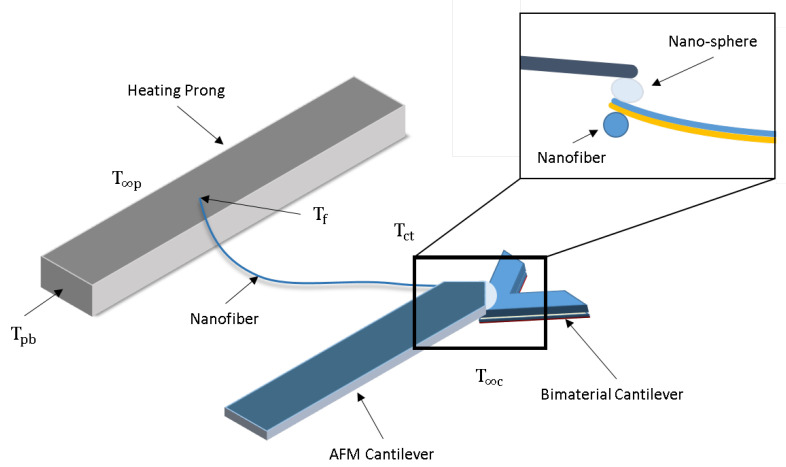
Design and model of the AFM utilizing Probe-to-Probe technique to determine the temperature boundary conditions of nanostructured materials [27].

After the creation of the nanofibers, a technique to measure these single polymer nanofibers is needed. In this work, the novel Probe-to-Probe method uses two cantilevers in forced actuation, as shown in Figure 1. This design focuses on a mechanical system rather than optical/vacuum methods utilized by Shen [12] et al. and Canetta [26] et al. from previous single polymer nanofiber measurement systems. The reason to create such a design is to mitigate complexity in measuring polymer nanofibers by joining two independent heating and testing subsystems. It allows an underlining cantilever and nanofiber sample to be easily loaded into a traditional atomic force microscope (AFM) cantilever without modification or cost.

The complete system utilizes an AFM, constructed loading sample plate with a (Cu) heating prong, along with tipless (Si) and bimaterial (Au/Si${}_{3}$N${}_{4}$) cantilevers tipped with a micro-sphere of radius 15 μm. The heat source is a ZENY 862D+ soldering iron with controlled temperature and is transferred sequentially through the sample plate, Cu heating prong, nanofiber, and lastly to the tip of the bimaterial cantilever. The competing thermal expansion of the Au and Si${}_{3}$N${}_{4}$ in the bimaterial cantilever exerts a force on the tipless AFM cantilever (AFM Workshop, Hilton Head Island, SC, USA) which is recorded by a 1 mW sensing laser (AFM Workshop, Hilton Head Island, SC, USA). From one-dimensional natural beam deflection theory and Hooke’s law, this deflection is used to obtain the temperature output, temperature at the cantilever tip ($T_{ct}$), from the nanofiber at the tip of the bimaterial cantilever. In congruence, the temperature of the nanofiber ($T_{f}$) measured at the heating prong is obtained through the use of thermocouples. The thermal conductivity of the nanofiber ($k_{f}$) can be characterized after determining the heat flux through the appended nanofiber.

## 2. Experimental Procedure

**Figure 2:**
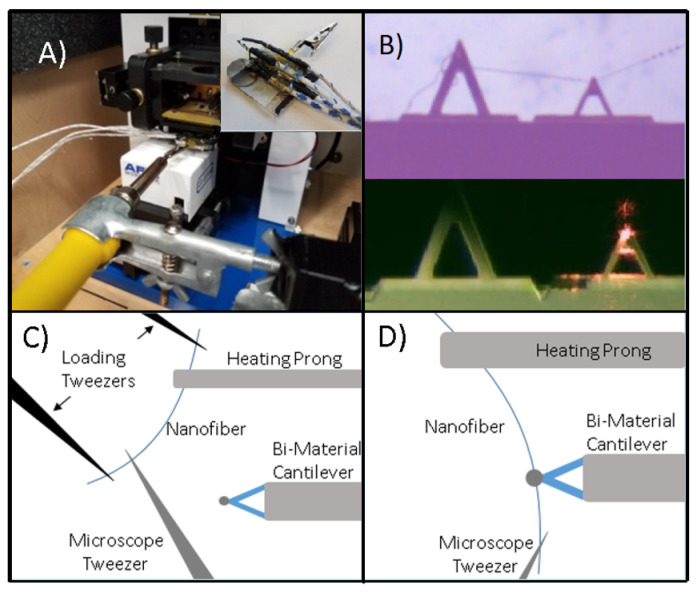
Experimental setup of (**A**) AFM sample plate when inserted into the AFM. Inset shows thermocouple positions on the sample plate. (**B**) Microscope image of a nanofiber appended to the bimaterial cantilever via van der Waals forces (top) and the calibration image of the cantilevers in the AFM (bottom). (**C**) Collecting the nanofiber from the electrospinning apparatus for appendage to the measurement apparatus. (**D**) Nanofiber appending itself to the bimaterial cantilever via van der Waals forces [27].

To appropriately measure the thermal properties of polymer nanofibers, a special AFM compatible sample plate needs to be prepared (Inset of Figure 2A). The sample plate is created by cutting a standard 1”× 3” microscope slide to one third of its original length (1”× 1”) and bonding it to a 90AFM Sample Mount to serve as a raised ledge for the bimaterial cantilever. A small strip of aluminum is placed beside the ledge to provide a surface level to the cantilever for the heating prong. PELCO Tabs are used to attach a steel disk to the sample plate to ensure it is properly held in place by the AFM’s magnetic platform. To act as the heating prong, a spade terminal connector (Neiko tools, Fujian, China) is held from the back end by an alligator clip, which is then heated by a soldering iron to serve as the heat source for the heating prong. Finally, four k-type thermocouples are attached to the sample plate, as shown in Figure 2A. One pair is to record the temperatures at the base of the heating prong ($T_{pb}$) and the temperature of the heating prong at the base of the nanofiber. The second pair is to record the ambient above the heating prong ($T_{\infty p}$), and the ambient above the attached cantilever ($T_{\infty c}$).

Obtaining the nanofibers is done through an electrospinning method [28,29]. Electrospinning can consistently produce nanofibers from many different polymers, but for the experiments run in this paper, polycaprolactone (PCL) nanofibers are used. The PCL nanofibers are formed by 10% weight/volume solution of PCL and acetone. After fully dissolved, the solution is loaded into a syringe with a 20AWG needle. This syringe is further inserted into a syringe pump, supplying the solution at a rate of 1 mL/min. The needle tip is attached to the positive lead of a high voltage power supply with the collector being grounded. A voltage differential of 10 kV is applied between the needle and the collector, and a tip to target distance of 9 cm is used to produce the most appropriate PCL nanofibers. The nanofiber collector consists of two parallel steel rods arranged perpendicularly to the needle [30,31]. The orientation of the apparatus allows for nanofibers to be spun in a fashion that they span the two parallel rods, allowing for the simple collection of an individual nanofiber. After the nanofibers are spun, they are collected, measured for dimensions [32], and appended to the sample plates, as seen in Figure 2A.

The nanofiber is collected from the electrospinning apparatus using a set of loading tweezers. The prongs of both of the heating prong and a microscope tweezer (a single prong of a surgical tweezer) are coated in UV glue to ensure proper bonding between the nanofiber and both the heating prong and the microscope tweezer. The single nanofiber is carefully collected from the electrospinning apparatus, the ends outside of the prongs are cut using a soldering iron to avoid stretching the nanofiber, which is possible due to PCL’s low melting temperature of 60 ${}^{\circ }$C. The nanofiber is then brought to the microscope.

A calibrated 10× OMax microscope (Chicago, IL, USA) is used to aid in appending a single nanofiber. The microscope’s XY-axis slide is removed in favor of a held positioning screw. The alligator clip of the sample plate is clamped to the screw, in a fashion that aligns well in the view under the microscope camera. As seen in Figure 2C, a single prong of a pointed tweezer is used to position and align the other end of the nanofiber.

Under the microscope, one end of the nanofiber is attached to the heating prong, held in place by the surface tension of the glue. The other end of the nanofiber is manipulated using a MaXYZ-60L XYZ stage that is mounted to a 3D printed base that allows it to rotate about a center point aligned with the tipless AFM cantilever. When the nanofiber is brought close enough to the tipless cantilever purchased from NanoandMore USA, the nanofiber attaches itself to the tipless cantilever via van der Waals forces, as shown in Figure 2B. A soldering iron is used to cut the outside end of the nanofiber.

The tipless Si cantilever is loaded into the AFM as for typical use. The sample plate is then slid onto the stage with the alligator clip mouth facing out of the AFM, underneath the AFM cantilever, as shown in Figure 2A. A stand and clamp is used to hold the soldering iron. The soldering iron is brought level with the AFM stage, and fixed to the alligator clip. The sample plates’ four thermocouples are then plugged into a LabView DAQ system.

## 3. Results

To ensure that the system is responding properly, the deflection of the AFM cantilever is measured in the unit of V/nm. Due to the non-traditional use of the AFM in this measurement, separate calibrations scans were performed to ensure the response of the tipless cantilever at higher than normal ambient temperatures. The maximum ambient temperatures conducted within these experiments only reached 27 ${}^{\circ }$C.

The voltage and deflection are recorded by two separate recording methods. Process 1 is to record the photodiode’s voltage output whereas process 2 records the natural deflection, via a held constant forced deflection, zeroed voltage. Using the AFM Workshop software displaying both these graphs as V/${}^{\circ }$C and nm/${}^{\circ }$C, respectively, provides deflection in the unit of V/nm. To measure V/${}^{\circ }$C, the AFM cantilever is held just above the sample plates’ bimaterial cantilever and heated. To measure deflection in nm/${}^{\circ }$C, the AFM cantilever is held in forced contact on the base of the bimaterial cantilever, zero voltage displacement, and heated; this provides the natural deflection of the cantilever.

The AFM cantilever does respond to small temperature variations during a system scan. Once the scan is completed and temperature is turned off, one notices that, over time, the cantilever reverts back to its original state via the photodiode in real time, which does not require a set scan to display.

The calibrated recorded values are compared to their theoretical generated values. Both theoretical results showcase how each cantilever (bimaterial or AFM-tipless) reacts under this novel Probe-to-Probe system, meaning which cantilever is actually causing the deflection/forcing seen. Referencing natural beam deflection [33,34] and Hooke’s law, and utilizing each cantilever’s properties with the recorded temperatures from calibration, one can obtain the theoretical values for this calibration step.

**Figure 3:**
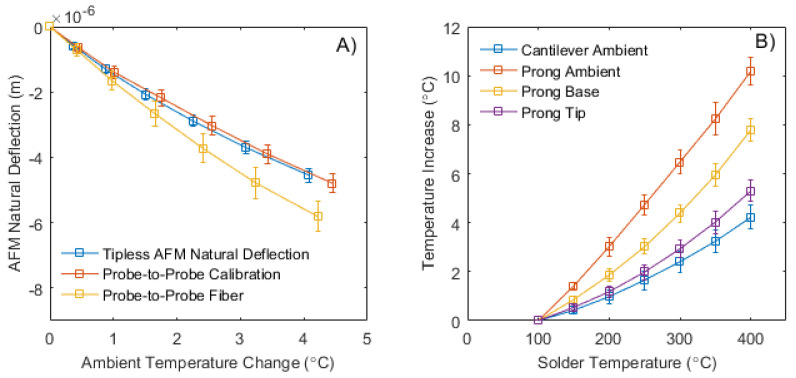
(**A**) Response of AFM cantilever with increasing ambient temperature during calibration and experimental scans. (**B**) Appended nanofiber temperature recordings at appended thermocouple positions ($T_{\infty c}$, $T_{\infty p}$, $T_{pb}$, and $T_{f}$) with increasing input solder temperature [27].

After recording deflection and temperature values once a nanofiber was appended, the recorded data must be subtracted from the recorded calibrated ones. These calibrated data are to provide true deflection versus change in ambient temperature solution as seen in Figure 3A. Figure 3B provides the averaged temperatures recorded by the sample plate’s thermocouples. Using these deflections after comparing them to the calibrated values and the averaged thermocouple data, $T_{f}$ and $T_{ct}$ can be determined [27].

When reviewing Table 1, it is noted that the ambient temperature over the cantilevers is similar to the input tip temperature of the bimaterial cantilever. It shows that there is very limited heat conducted to the tip of the bimaterial cantilever, if none at all, or the use of ambient heating is overcoming any heat transfer. The thermocouple position is consistent and accurate between scans when adjusted to the system; however, it is possible that this position is not ideal, and ambient recordings varies. All temperature data are recorded as an increase in temperature from its initial point at 100 ${}^{\circ }$C.

## 4. Discussion and Conclusions

Future work around this study is currently being considered with plans to record thermal flux through the polymer nanofiber in order to fully characterize the thermal conductivity of a single polymer nanofiber. Obtaining the thermal conductivity of a single nanofiber is a two step process. The first process is to run a calibration scan to record deflections from the AFM when it contains no nanofiber. The AFM system contains only the heating prong, bimaterial and tipless cantilevers. This scan mitigates any external temperatures and vibrations that occur before the nanofiber is appended. The second process is to apply the nanofiber and record the AFM deflections and temperatures. Working back from beam deflection theory determines the temperature at the tip of the bimaterial cantilever that causes this deflection. Fourier’s law can be used to determine the thermal conductivity of an individual nanofiber, such as $q_{f}=-k_{f}\frac{\Delta T}{\Delta x}$, where ΔT is the differential between $T_{f}$ and $T_{ct}$, 1.0766 ${}^{\circ }$C and Δx is the length of the individual nanofiber; here, 7.7 mm. Using the Probe-to-Probe method, the difference between the appended nanofiber temperatures is determined, taking the recorded temperature values from Table 1. Given the testing parameters and constraints of the proposed Probe-to-Probe measurement system, we experimentally demonstrated a recorded high thermal conductivity of 103.67 W/(m·K) for a single PCL nanofiber with a diameter of 750 nm (refer to Chapter 5 in [27]).

Considering the melting point of PCL (around 60 ${}^{\circ }$C), we have taken this in account for determining the temperature limit of the heating prong while making sure its maximum temperature is below 60 ${}^{\circ }$C in this work. Otherwise, the nanofibers will melt. In Table 1, all temperature data are recorded as an increase in temperature from its initial point at 100 ${}^{\circ }$C. They are all less than the melting point temperature of the polymer even when the solder temperature is at maximum 400 ${}^{\circ }$C. The diameter of the fibers may influence the thermal conductivity of the nanofiber thin films. Zhong et al. showed a size dependence with higher thermal conductivities for fibers with smaller diameter, which is possibly due to better chain alignment in smaller fibers [14]. However, Ma et al. did not state a clear relation between the thermal conductivity and the size of nanofibers [13]. The diameter of nanofibers depends on the electrospinning voltage and weight/volume ratio of the solution. Overall, a better alignment of nanofibers enhances the thermal conductivity of nanofiber-supported thin films. However, the size dependence on a single nanofiber’s thermal conductivity needs to be further explored.

In this work, it is demonstrated that an AFM has the capability to assist in measuring the thermal conductivity of an individual nanofiber using this novel Probe-to-Probe method. This is completed entirely through proper preparation of the nanofiber test sample with no modifications being made to the AFM itself. The Probe-to-Probe method accurately measured the temperature boundary conditions about a PCL nanofiber with a heating prong temperature of 400 ${}^{\circ }$C. This method supports previous work showing enhanced thermal conductivity in electrospun polymer nanofibers by demonstrating increased heat conducted to the AFM tip end of the polymer nanofiber. The Probe-to-Probe method outlined here is an effective tool in establishing baseline measurements for determining the enhanced thermal conductivity of polymer nanofibers.

## Figures and Tables

**Table 1:** Recorded temperature increase with appended nanofiber.

Solder Temp (${}^{\circ }$C)	$T_{\infty p}$ (${}^{\circ }$C)	$T_{\infty c}$ (${}^{\circ }$C)	$T_{\mathrm{pb}}$ (${}^{\circ }$C)	$T_{f}$ (${}^{\circ }$C)	$T_{\mathrm{ct}}$ (${}^{\circ }$C)
100	0	0	0	0	0
150	1.4151	0.42494	0.85987	0.54005	0.42494
200	3.0192	0.97729	1.8575	1.1809	0.97729
250	4.7307	1.6543	3.016	1.986	1.6543
300	6.4784	2.4063	4.389	2.9301	2.4063
350	8.2314	3.2395	5.9458	4.026	3.2395
400	10.18	4.2176	7.7851	5.2942	4.2176
